# In silico and in vivo anti-malarial investigation on 1-(heteroaryl)-2-((5-nitroheteroaryl)methylene) hydrazine derivatives

**DOI:** 10.1186/s12936-020-03269-7

**Published:** 2020-06-29

**Authors:** Azar Tahghighi, Seyed-Mahdi Mohamadi-Zarch, Hamzeh Rahimi, Mahya Marashiyan, Naseh Maleki-Ravasan, Ali Eslamifar

**Affiliations:** 1 Medicinal Chemistry Laboratory, Department of Clinical Research, Pasteur Institute of Iran, Tehran, Iran; 2 Malaria and Vector Research Group (MVRG), Biotechnology Research Center (BRC), Pasteur Institute of Iran, Tehran, Iran; 3 Department of Physiology, Faculty of Medicine, Shahid Sadoughi University of Medical Sciences, Yazd, Iran; 4 Molecular Medicine Department, Biotechnology Research Center, Pasteur Institute of Iran, Tehran, Iran; 5 Department of Parasitology, Pasteur Institute of Iran, Tehran, Iran; 6 Department of Clinical Research, Pasteur Institute of Iran, Tehran, Iran

**Keywords:** Anti-plasmodial activity, Docking study, Quinoline, Quinazoline, *Plasmodium berghei*, Histopathology, Pharmacodynamics

## Abstract

**Background:**

Resistance of *Plasmodium falciparum* against common anti-malarial drugs emphasizes the need of alternative and more effective drugs. Synthetic derivatives of 1-(heteroaryl)-2-((5-nitroheteroaryl)methylene) hydrazine have showed in vitro anti-plasmodial activities. The present study aimed to evaluate the molecular binding and anti-plasmodial activity of synthetic compounds in vivo.

**Methods:**

The molecular docking was used to study the binding of compounds to haem and *Plasmodium falciparum* lactate dehydrogenase (PfLDH). Acute toxicity of the synthetic compounds was evaluated based on the modified up & down method. The anti-plasmodial activity of the compounds was conducted by the two standard tests of Peters’ and of Rane, using chloroquine-sensitive *Plasmodium berghei* in mice. Also, the toxicity to the internal organs of mice was evaluated on the seventh day after the treatment in addition to the histopathology of their liver. Compound **3** that showed high activity in the lowest dose was selected for further pharmacodynamic studies.

**Results:**

According to the docking studies, the active site of PfLDH had at least four common residues, including Ala98, Ile54, Gly29, and Tyr97 to bind the compounds with the affinity, ranging from − 8.0 to − 8.4 kcal/mol. The binding mode of ligands to haem revealed an effective binding affinity, ranging from − 5.1 to − 5.5 kcal/mol. Compound **2** showed the highest  % suppression of parasitaemia (99.09%) at the dose of 125 mg/kg/day in Peters’ test. Compound **3**, with 79.42% suppression, was the best in Rane’s test at the lowest dose (31 mg/kg/day). Compound **3** was confirmed by the pharmacodynamic study to have faster initial parasite elimination in the lowest concentration. The histopathology of the livers of mice did not reveal any focal necrosis of hepatocytes in the studied compounds.

**Conclusions:**

The docking studies verified *Pf* LDH inhibition and the inhibitory effect on the haemozoin formation for the studied compounds. Accordingly, some compounds may provide new avenues for the development of anti-malarial drugs without liver toxicity, although further studies are required to optimize their anti-plasmodial activity.

## Background

Malaria is one of the most important infectious diseases that threatens half of the world’s population. The world malaria report 2019 estimated that there were 228 million cases of malaria in 2018 causing 405,000 global deaths [1]. With 272,000 deaths, children less than 5 were the most vulnerable group worldwide. In Africa, 213 million people were affected by malaria, which made it the most vulnerable continent in 2018 [1]. Worldwide, there was significant reduction of malaria cases during 2015–2017, but there is a major challenge to thoroughly eliminate malaria in many countries by 2030 [2].

**Fig. 1:**
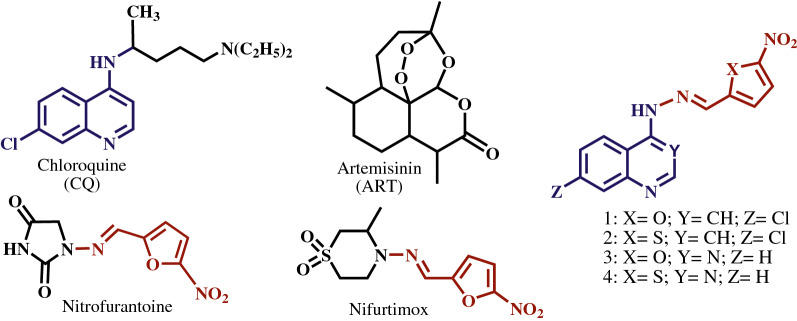
References and experimental compounds

As shown in Fig. 1, chloroquine (CQ) and artemisinin (ART) derivatives are the two main classes of anti-malarial drugs [3]. However, repetitive and inappropriate use of CQ caused drug resistance of malaria parasites. The decreased susceptibility of parasites to ART in the Greater Mekong Subregion (GMS)—Laos, Myanmar, Cambodia, Thailand, and Southern Vietnam—may also extend to other endemic areas [4, 5]. The epidemiological evidence predicts the “tsunami” of ART resistance in the world, called “super malaria”. In this situation, subsequent treatment failures with artemisinin-based combination therapy (ACT) have raised concerns about the loss of the only highly-effective treatment currently available to treat malaria [6].

Several research groups worked on the development of novel anti-malarial drug pipelines with some chemical modification on the current drugs [7, 8]. Moreover, natural products are noteworthy as new sources of anti-plasmodial [9, 10]. Therefore, it is necessary to use new, effective, low-cost, safe, and affordable alternative anti-malarial agents [11].

Given the importance of the subject, new analogs of quinoline anti-malarial drugs were designed and synthesized by integrating the important biological rings of quinoline and quinazoline with the active fragment of (5-nitroheteroaryl)methylene hydrazine, inspired by antimicrobial drugs, nitrofurantoin, and nifortimox (Fig. 1). Nitrofurantoin is an antibiotic that is used to treat bladder infections and prophylaxis of infection [12]. Nifurtimox is an effective medication in the treatment of sleeping sickness and Chagas disease [13]. In the last study [Tahghighi et al. pers. commun], *Plasmodium falciparum* lactate dehydrogenase assay (PfLDH assay) showed that the synthetic compounds were effective against CQ-sensitive (3D7) and CQ-resistant (K1) *P. falciparum* strains. Also, haemozoin formation in 3D7 and (K1) *P. falciparum* strains with synthetic compounds was measured via β-haematin assay [Tahghighi et al. pers. commun]. The results of β-haematin and PfLDH assay affirmed the potency of the anti-malarial activity of these compounds. In the present study, the binding of the synthetic compounds to haem and PfLDH was investigated by the molecular docking. Also, given the importance of in vivo tests, the anti-plasmodial activity of the compounds was assessed by the Peters’ and the Rane’s tests in mice inoculated with *Plasmodium berghei* (ANKA strain). The histological changes in the liver mouse malaria model were evaluated on the seventh day after the treatment. Also, compound **3** with high activity in the lowest dose on 5th day after the treatment was chosen for further pharmacodynamic studies.

## Methods

The materials and chemical reagents were purchased from Sigma-Aldrich Company (USA). Compounds **1** to **4** were prepared in line with previous research [Tahghighi et al. pers. commun]. The binding of ligands to haem and PfLDH were studied based on the molecular docking via AutoDock tool. The female BALB/C mice were purchased from the Pasteur Institute of Iran. Chloroquine sensitive *P. berghei* (ANKA strain) was obtained from the department of Medical Parasitology & Mycology, School of Public Health, Tehran University of Medical Sciences, Iran.

### Receptor structure preparation and generation of grid box for docking studies

Molecular docking is a worthwhile method to explore the protein and ligand interactions at the molecular level [14]. This method was used to study the binding of ligands to haem and *P. falciparum* L-lactate dehydrogenase (PfLDH). For molecular docking experiment, the 3D structure of the L-lactate dehydrogenase and haem were taken from the Protein Data Bank (PDB ID 1LDG and 3P5Q). To this end, the receiver structure was prepared using the AutoDock toolkit. Also, the missing atoms were added, residues were assigned, and the AutoGrid parameter file was adjusted via AutoDock tools [15]. The L-lactate dehydrogenase active site was selected as the ligand binding site [16]. The size of the docking grid for L-lactate dehydrogenase and haem were X = 32 A˚ Y = 30 A˚ and Z = 32 A˚ and X = 14 A˚, Y = 10 A˚ and Z = 14 A˚, respectively. The grid spacing was adjusted on 1 Å.

### Ligand molecule preparation

The ligand molecules, drawn by Marvin 19.10 were subsequently optimized via HyperChem 8.0 software [17].

### Molecular docking study

The ligand molecules were docked in the selected binding pocket via Smina AutoDock. Smina is a version of AutoDock Vina, that focuses on the improvement of scoring and energy minimization [18]. Then, the crystal structure of receptors in complex with ligands was analysed via LigPlot^+^ software [19].

### In vivo anti-malarial assay

#### Acute toxicity of the compounds in mice

Before in vivo study, the toxicity of the compounds was assessed with some modifications by Dixon’s ‘up and down’ method in BALB/c mice [20]. In the first phase, 500 mg/kg of compounds were administered intraperitoneally to two mice and observed for any signs of toxicity and mortality at 24 h. When a mouse died, the concentration was reduced to half, the test was repeated, and the mice were monitored for toxicity and mortality for 10 days. In the second phase, the test was repeated with 5 mice, and if they survived, the highest non-toxic dose would be determined for the Rane’s and the Peters’ tests (125 mg/kg).

#### Parasite inoculation

At first, two female BALB/C mice were infected intraperitoneally from the frozen stock of CQ-sensitive *P. berghei* (ANKA strain). Then, four and six mice were infected by continuous intraperitoneal passage. Next, the blood was diluted with PBS, and finally the experimental mice were infected with an inoculum of 1.5 × 10^7^ of parasitized erythrocytes intraperitoneally.

#### Schizonticidal effect in early infection (Peters’ test)

The suppressive test was conducted in accordance with Peters’ method [8, 21]. The experimental mice were maintained under the standard conditions based on the international guidelines for 10 days [22]. Seventy-five female BALB/C mice (weight 16–20 g) were grouped into fifteen groups. They were inoculated intra-peritoneally (i.p.) with 1.5 ×10^7^ infected erythrocytes of CQ-sensitive *P. berghei* in a saline (200 μl) on the first day (D0) of the test. The test compounds were solubilized in a mixture of DMSO, sesame oil, and ethanol (a ratio of 1:2:0.05) pre-diluted in sesame oil for preparation of different dose (31, 62, and 125 mg/kg). The treatments began within 3 h post-inoculation of mice with the parasite (D0) and proceeded intraperitoneally for 4 days (D3). The tail blood smear was stained with 10% Giemsa in phosphate buffer (pH 7.2) on the fifth day (D4). The parasitaemia level was determined by counting the parasitized red blood cells on at least 2000 red blood cells by microscope at 100×. The  % suppression of parasitaemia was calculated by comparing the % parasitaemia between the test mice and infected controls. The chloroquine diphosphate (25 mg/kg), oil and DMSO (7%) were applied as positive and negative controls. For all the groups, mortality was monitored daily and the mean survival time was recorded to evaluate the efficacy of anti-malarial activity of the compounds. During the treatment, the body weight of each mouse was measured before infection (D0) and after the treatment (D4). Also, the internal organs (spleen, liver, and kidney) were evaluated on the seventh day (D6) of the treatment after the dissection of the mice.

#### Schizontocidal activity in established infection (Rane’s test)

The curative test was conducted according to the Rane’s test (as described Ryley and Peters) [23]. The animal housing, infecting, and dosing were done similar to Peters’ test, but the treatment began within 72 h after intraperitoneally inoculation of mice with the parasite (D3), allowing parasitaemia to establish and then continue for 4 days (D6). The tail blood smears were stained with 10% Giemsa in phosphate buffer (pH 7.2) on the 8th day (D7). The parasitaemia level and the % suppression of parasitaemia, mortality, mean survival time, and body weight were determined and recorded in line with Peters’ test.

#### Histopathological study of liver tissue

Histopathological studies were conducted based on the standard method [24]. After the dissection on the seventh day (D6) after the treatment, mice’s livers were fixed in 10% formalin solution. Then, the middle lobe of livers was severed, and molds of fresh paraffin were made of tissue with a thickness of 5 µm. The sections were stained with haematoxylin–eosin (H&E) and analysed by light microscope. The slides were examined under 40× objective lens and the image was saved in JPEG files. As described by Suzuki et al., the sections were scored from 0 to 4 for sinusoidal congestion, vacuolization of hepatocyte cytoplasm, and parenchymal single cell necrosis [25].

### In vivo pharmacodynamic study of compound 3

The pharmacodynamic study was conducted using the rodent malaria *P. berghei* ANKA model in female BALB/c mice (weight 18–22 g) (for each group, n = 5) [26]. The mice were inoculated intraperitoneally with 1.5 × 10^7^ parasitized erythrocytes in a saline (200 μl) on the first day (D0). Compound **3** was administered by oral gavage (125 mg/kg) and i.p. (31 mg/kg, and 125 mg/kg) after 3 h of post-inoculation of mice with the parasite (D0) and followed for 4 days (D3). Control mice were received oil/DMSO (7%).  % parasitaemia was investigated every 2 days until the parasitaemia > 35%. The body weight of all the mice was recorded on D0 until the end of test every 2 days. The average survival time of the mice was monitored daily.

### Statistical analysis

The data were analysed using SPSS (IBM SPSS Statistics 22). Then one-way ANOVA was used to test the statistical differences for three doses within a group, followed by Tukey’s test for multiple comparisons. *P*-values < 0.05 were considered significant in all tests.

## Results

### In silico investigation of molecular interactions

**Fig. 2:**
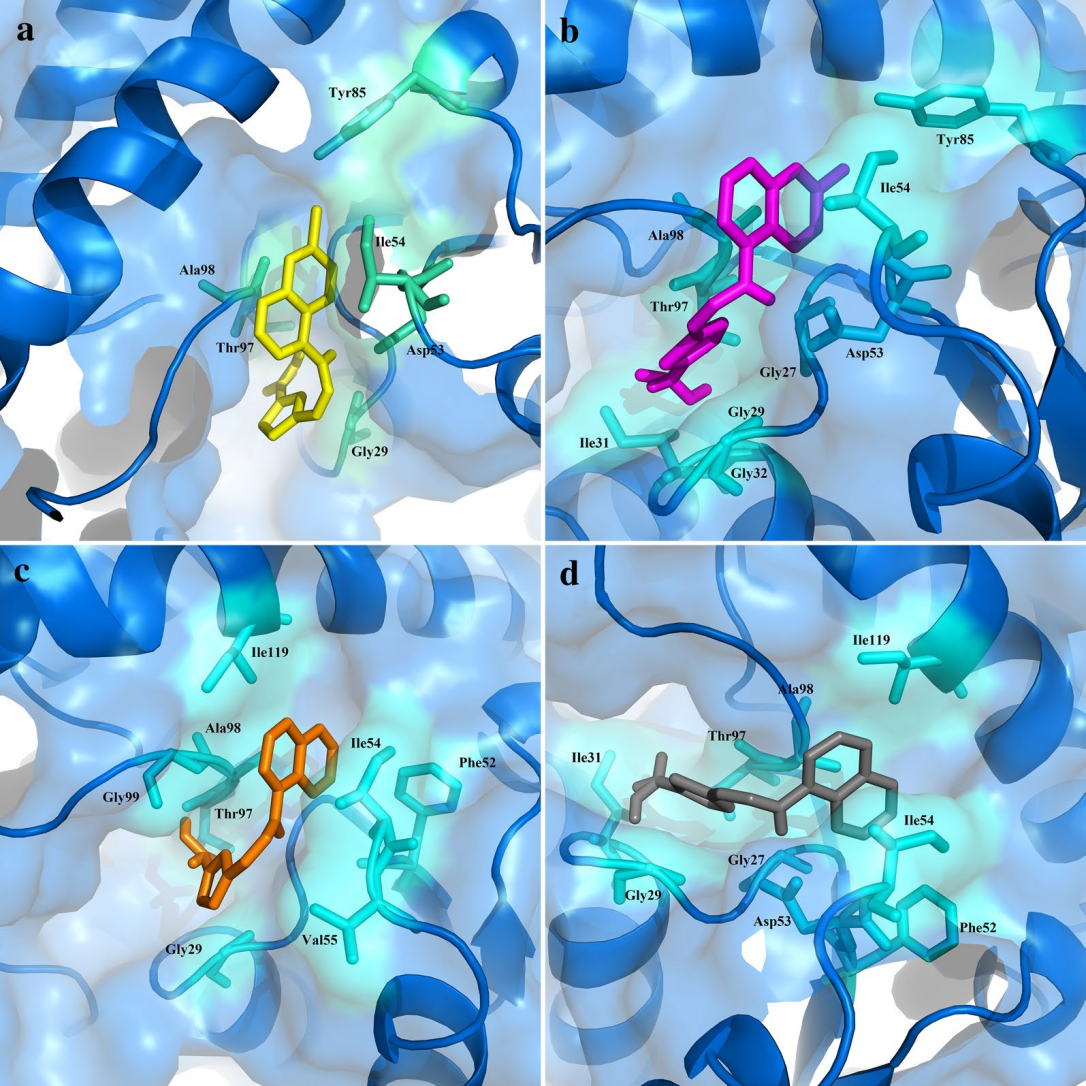
Residues involved in the L-lactate dehydrogenase and ligands interactions. Surface representation of L-lactate dehydrogenase was shown in blue. **a** Interacting residues of L-lactate dehydrogenase with ligand1 in yellow; **b** Interacting residues of L-lactate dehydrogenase with ligand2 in magenta; **c** Interacting residues of L-lactate dehydrogenase with ligand3 in orange; **d** Interacting residues of L-lactate dehydrogenase with ligand4 in gray

**Fig. 3:**
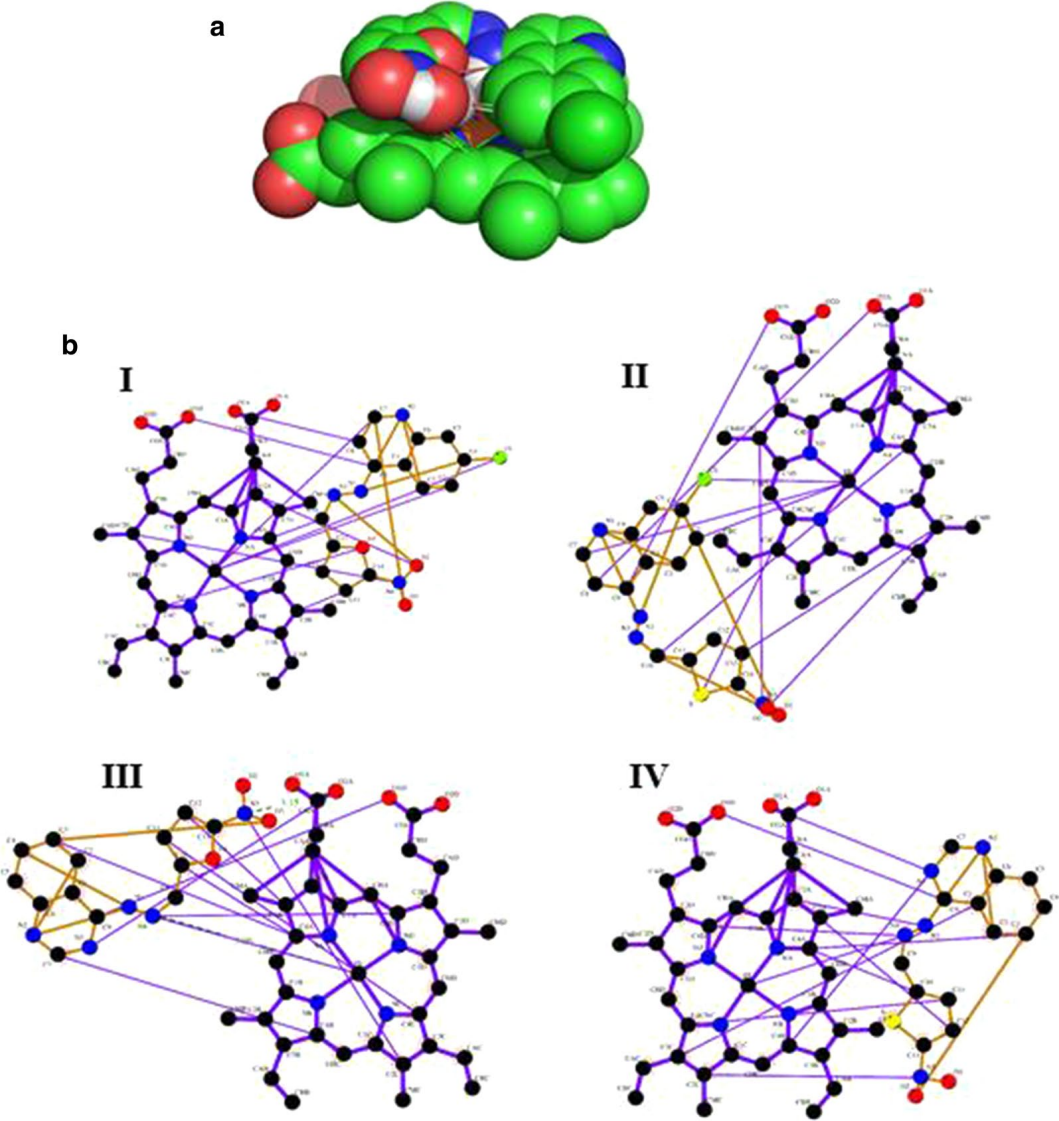
Representation of interaction of haem with ligands. **a** Illustration of haem in complex with ligand; **b** The Ligplot analysis for haem and ligands interaction. Heme-lig1, lig2, lig3 and lig4 complexes were shown in I, II, III, and IV, respectively. Haem was shown in magenta and ligands in orange. Green dashed lines were illustrated hydrogen bonds

Marvinsketch was used to draw ligands molecule and optimize with HyperChem software. Shown in Fig. 1, the ligands were docked at haem and the active site of the l-lactate dehydrogenase in AutoDock Smina to generate the best possible conformations with the lowest binding energies. The molecular docking results demonstrated that l-lactate dehydrogenase complexes with lig1, lig 2, lig 3, and lig 4 with the range of binding affinity as − 8.2 to − 7.4, − 8.0 to − 7.0, − 8.4 to − 7.4 and, − 8.2 to − 6.8 kcal/mol, respectively. These values for haem complexes were − 5.4 to − 4.7, − 5.1 to − 4.6, − 5.5 to − 4.9 and − 5.2 to − 4.8, respectively. Table 1 shows the results of the best binding affinities. The binding mode interactions were analysed using Ligplot software. Figure 2 shows the significant L-lactate dehydrogenase residues are involved in the interaction with ligands. Moreover, the molecular docking results showed that the binding pocket of ligands had at least four common residues, including Ala98, Ile54, Gly29, and Tyr97. Additional file 1: Fig S1 illustrates the binding mode interactions of complexes. To study the binding mode of ligands to haem, the best docked complexes were subjected to Ligplot^+^, the analysis that allows the identification of the ligand-receptor contacts. The atoms involved in ligand binding are shown in Fig. 3.

**Table 1:** Evaluation of best binding affinity of L-lactate dehydrogenase and haem with the studied ligands

Complexes	Best binding affinity to PfLDH(Kcal/mol)	Complexes	Best binding affinity to Haem (Kcal/mol)
PfLDH-lig1	− 8.2	Heme-lig1	− 5.4
PfLDH -lig2	− 8.0	Heme-lig2	− 5.1
PfLDH -lig3	− 8.4	Heme-lig3	− 5.5
PfLDH -lig4	− 8.2	Heme-lig4	− 5.2

### In vivo acute toxicity of the synthetic compounds

The examined mice at the dose of 125 mg/kg were normal showing no behavioral and physical changes after 10 days. Three test doses of the synthetic compounds were selected based on the highest non-toxic dose (125 mg/kg) for the anti-plasmodial activity evaluation in mice.

### Evaluation of schizonticidal activity in early infection for the compounds (1–4)

**Fig. 4:**
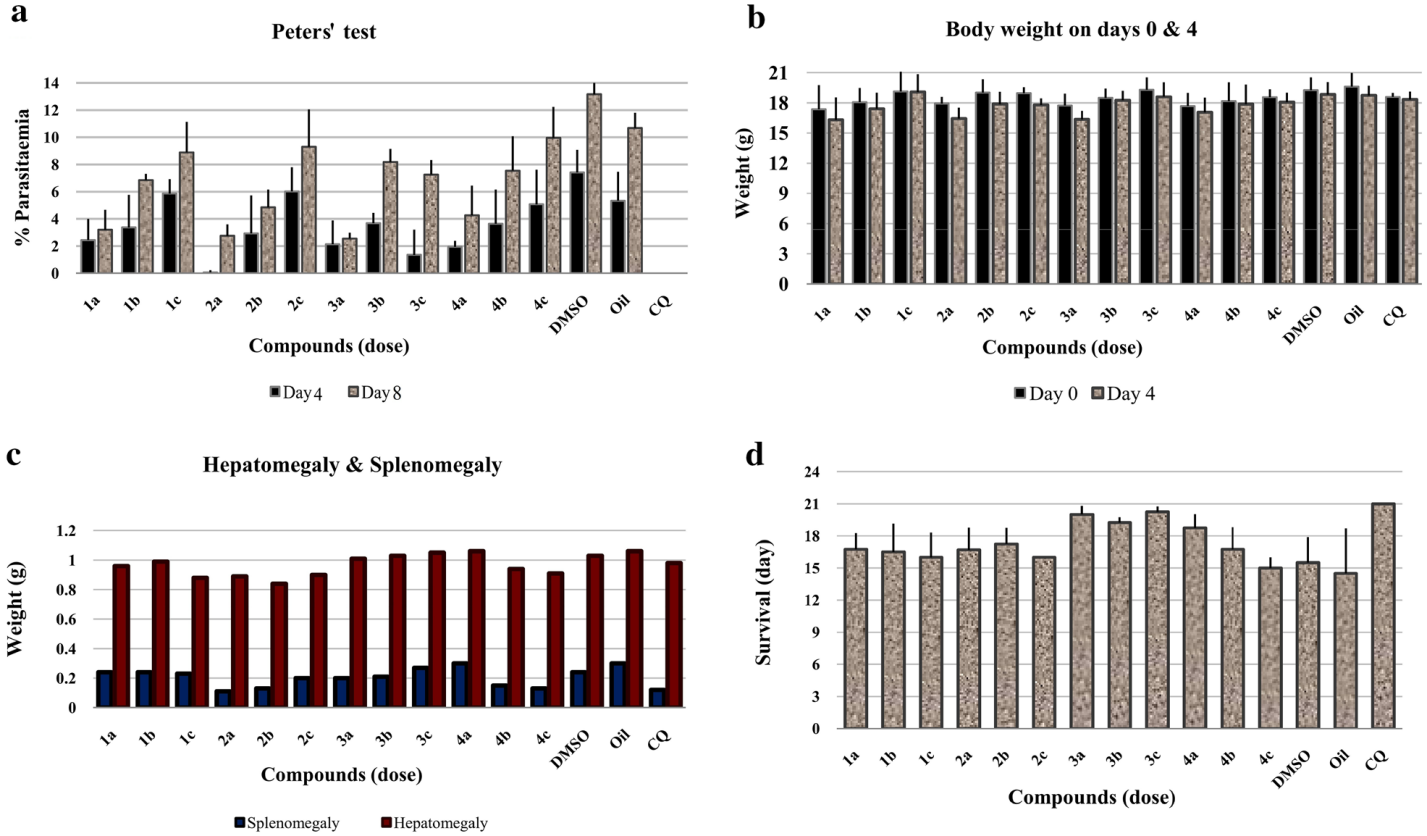
The anti-plasmodial activity of the synthetic compounds in early infection of mice (4-day suppressive test) at various doses (a: 125, b: 62, and c: 31 mg/kg). **a**  % Parasitaemia of infected mice on day 4 and 8; **b** Body weight of *P. berghei* infected mice on day 0 and 4; **c** Toxicity of internal organs on day 6; **d** Mean survival time

The mice that were treated with 125 mg/kg dose showed 61.69, 99.09, 66.50, and 69.39% chemo-suppressive anti-malarial activity for groups **1a** to **4a**, respectively. The group **3c** showed the most activity at lower dose (78.74%) (Table 2, Fig. 4a). Chloroquine showed 100% suppression with a dose of 25 mg/kg. The control groups died 2 weeks after the infection. Only CQ group survived until the end of the experiment (Day 21). The body weight on day 5 (D4) decreased compared with first day (D0) (Fig. 4b).

**Table 2:** Evaluation of average  % parasitaemia and  % suppression of parasitaemia in Peters’ test

Groups	Compounds	Dose (mg/kg)	Average % parasitaemia ± SD (Day 4)	% Suppression of parasitaemia (Day 4)	Average % parasitaemia ± SD (Day 8)	*P*-value
Oil	–	–	5.33 ± 2.12	–	10.68 ± 1.11	–
DMSO	–	–	7.41 ± 1.65	–	13.16 ± 1.47	–
CQ	–	25	0	100	0	–
1	**1a**	125	2.44 ± 1.56	61.69	3.20 ± 1.46	
	**1b**	62	3.37 ± 2.39	47.10	6.85 ± 0.45	< 0.05
	**1c**	31	5.86 ± 1.06	8.01	8.89 ± 2.25	
2	**2a**	*125*	*0.06 ± 0.17*	*99.09*	*2.75 ± 0.84*	
	**2b**	62	2.92 ± 2.79	54.16	4.85 ± 1.30	< 0.05
	**2c**	31	6.02 ± 1.79	5.49	9.30 ± 2.75	
3	**3a**	125	2.13 ± 1.75	66.50	2.55 ± 0.42	
	**3b**	62	3.68 ± 0.76	42.26	8.18 ± 0.97	< 0.05
	**3c**	*31*	*1.35 ± 1.86*	*78.74*	*7.25 ± 1.07*	
4	**4a**	125	1.95 ± 0.44	69.39	4.26 ± 2.17	
	**4b**	62	3.63 ± 2.51	43.01	7.54 ± 2.52	< 0.05
	**4c**	31	5.07 ± 2.54	20.41	9.96 ± 2.27	

The in vivo activities were evaluated against *Plasmodium berghei*

Italic values indicate the best compounds based on Peters’ test

*SD* standard deviation

Compared to the control groups, especially for **3a**–**c** and **4a** ones, a mild enlargement of the liver in the treated groups were observed after the dissection of the internal organs (Fig. 4c). The quantitative evaluation of the spleens of treated mice showed a considerable increase comparing to the CQ except **2a**–**b** and **4b**–**c** groups (Fig. 4c). The treatment groups survived longer than the control groups except group **4c** as shown in Fig. 4d. There was no significant change in the kidneys of treated groups.

### Evaluation of schizontocidal activity in established infection for the compounds (1–4)

**Fig. 5:**
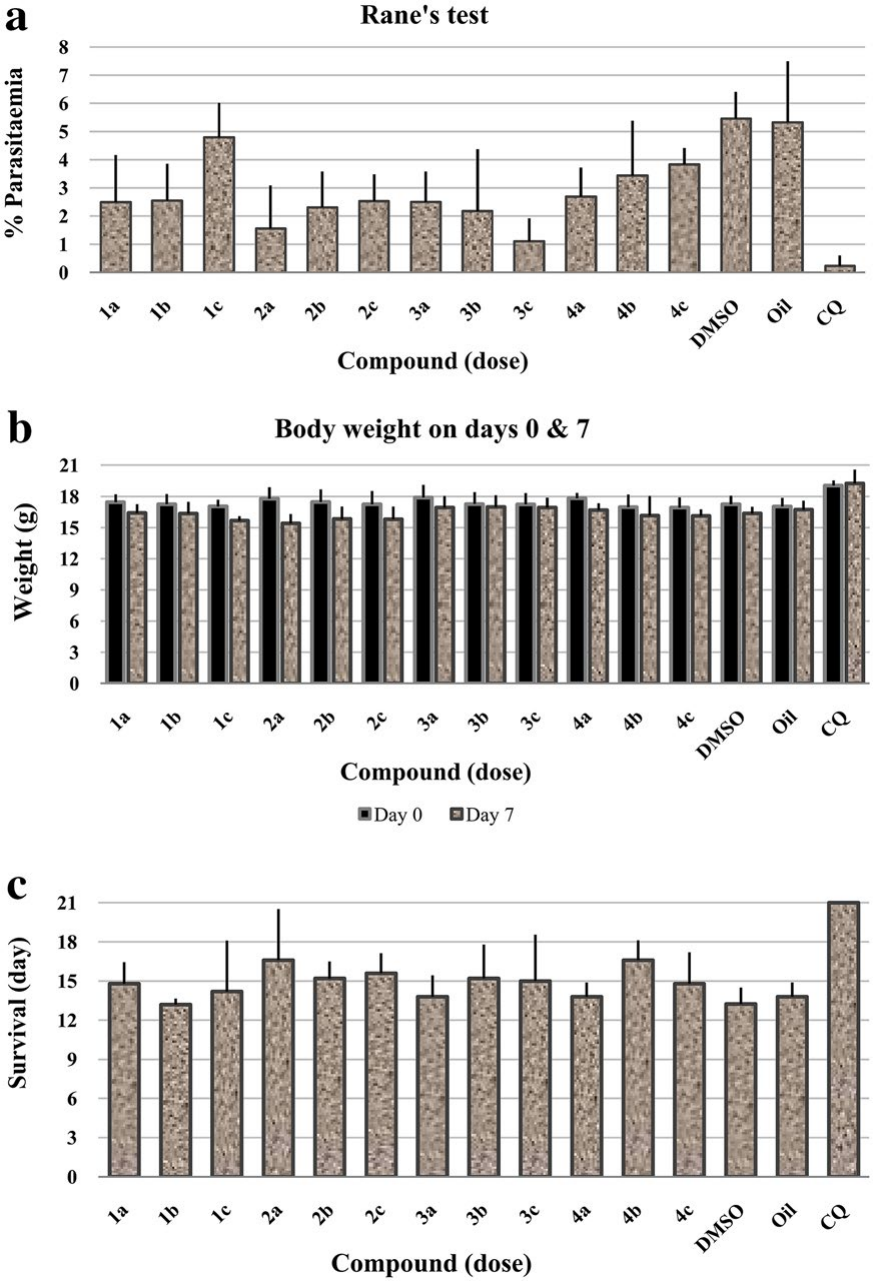
Anti-plasmodial activity of the synthetic compounds in establish infection of mice (Curative Test) at various dose (a: 125, b: 62, and c: 31 mg/kg). **a**  % Parasitaemia of infected mice on day 7; **b** Body weight of *P. berghei* infected mice on day 0 and 7; **c** Mean survival time

The mice treated with 125 mg/kg dose showed 53.75, 71.07, 53.61, and 50.08% chemo-suppressive anti-malarial activity for **1a** to **4a** groups, respectively. Similar to Peters’ test, the group **3c** showed the most activity at lower dose (79.42%) (Table 3, Fig. 5a). On day 8, the body weight (D7) decreased (Fig. 5b). The mice treated with the synthetic compounds significantly survived longer than the control groups except for **1b**, **3a** and **4a** groups, as shown in Fig. 5c. The mice, treated with the CQ, survived until 21 day after the treatment.

**Table 3:** Evaluation of average % parasitaemia and  % suppression of parasitaemia in Rane’s test

Groups	Compounds	Dose (mg/kg)	Average % parasitaemia ± SD (Day 7)	% Suppression of parasitaemia (Day 7)	CLogP	*P*-value
Oil	–	–	5.33 ± 2.17	–	–	–
DMSO	–	–	5.46 ± 0.94	–	–	–
CQ	–	25	0.21 ± 0.22	96.10	5.06	–
1	**1a**	125	2.49 ± 1.67	53.75		
	**1b**	62	2.55 ± 1.30	52.72	3.93	> 0.05
	**1c**	31	4.79 ± 1.22	11.11		
2	**2a**	*125*	*1.56 ± 1.53*	*71.07*		
	**2b**	62	2.31 ± 1.27	57.17	4.46	> 0.05
	**2c**	31	2.53 ± 0.95	53.05		
3	**3a**	125	2.50 ± 1.08	53.61		
	**3b**	62	2.72 ± 2.10	49.56	2.50	> 0.05
	**3c**	*31*	*1.11 ± 0.81*	*79.42*		
4	**4a**	125	2.69 ± 1.02	50.08		
	**4b**	62	3.44 ± 1.95	36.25	3.03	> 0.05
	**4c**	31	3.84 ± 0.58	28.87		

The in vivo activities were evaluated against *Plasmodium berghei*

Italic values indicate the best compounds based on Rane’s test

*SD* standard deviation

### Histopathological study of mice livers

**Fig. 6:**
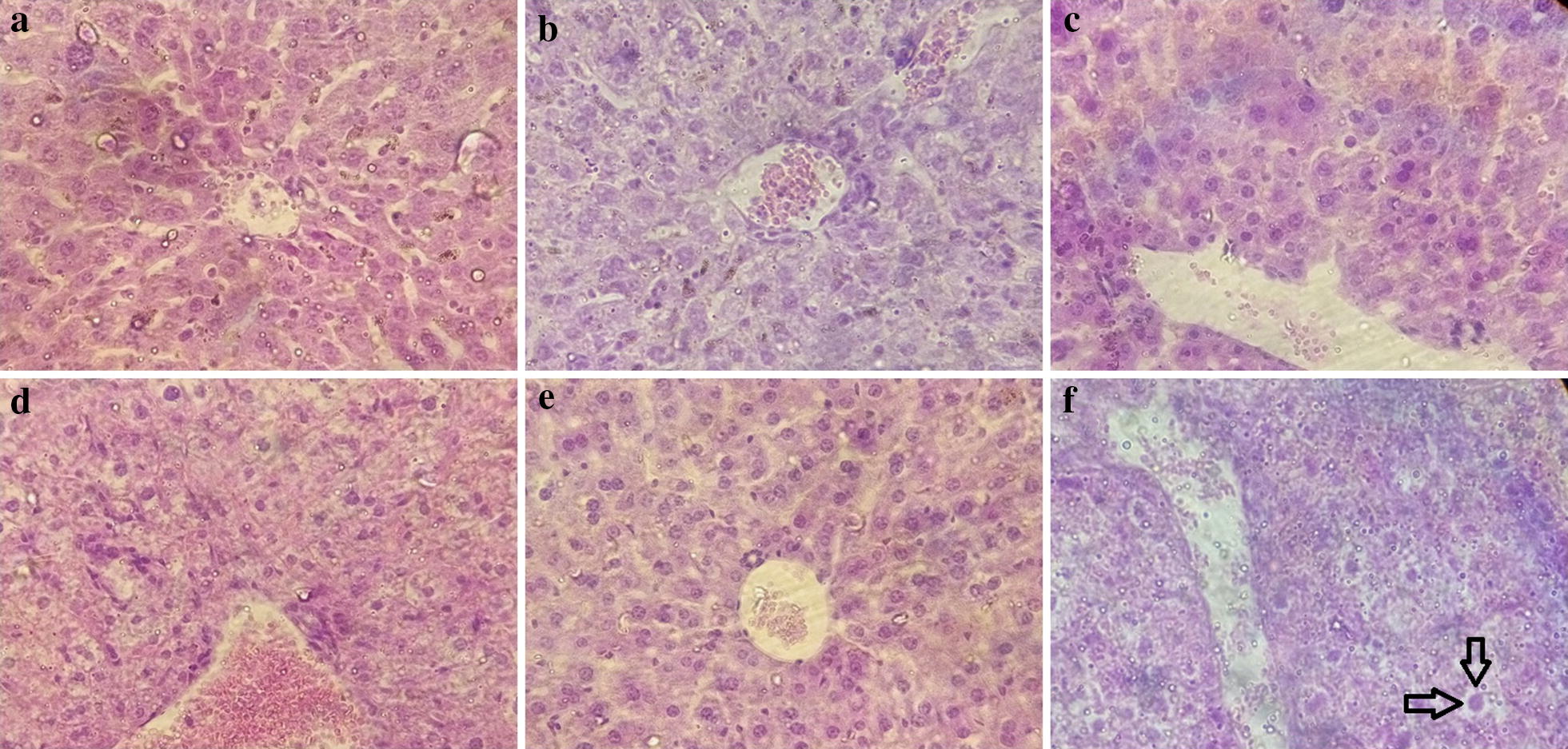
Liver segments of a mouse treated with **a** compound **1** (125 mg/kg); **b** compound **2** (125 mg/kg); **c** compound **3** (125 mg/kg); **d** Compound **4** (125 mg/kg); **e** oil, **f** CQ (25 mg/kg) on the 7th day. The arrows indicate single cell necrosis

The histopathological study of the treated mice’s livers with the synthetic compounds after 7 days of treatment was evaluated in higher studied dose (125 mg/kg) for Peters’ test. The liver of the mice did not show any damage whatsoever, including vacuolization, minimal congestion, and single-cell necrosis, while the mice that received CQ showed minimal sinusoidal animal congestion, vacuolization, and single cell necrosis (Table 4, Fig. 6).

**Table 4:** Histopathological evaluations of the liver

Compounds	Congestion	Vacuolization	Necrosis	Score
Oil	None	None	None	0
CQ	Minimal	Minimal	Single cell necrosis	1
**1**	None	None	None	0
**2**	None	None	None	0
**3**	None	None	None	0
**4**	None	None	None	0

### In vivo pharmacodynamic study of compound 3

**Fig. 7:**
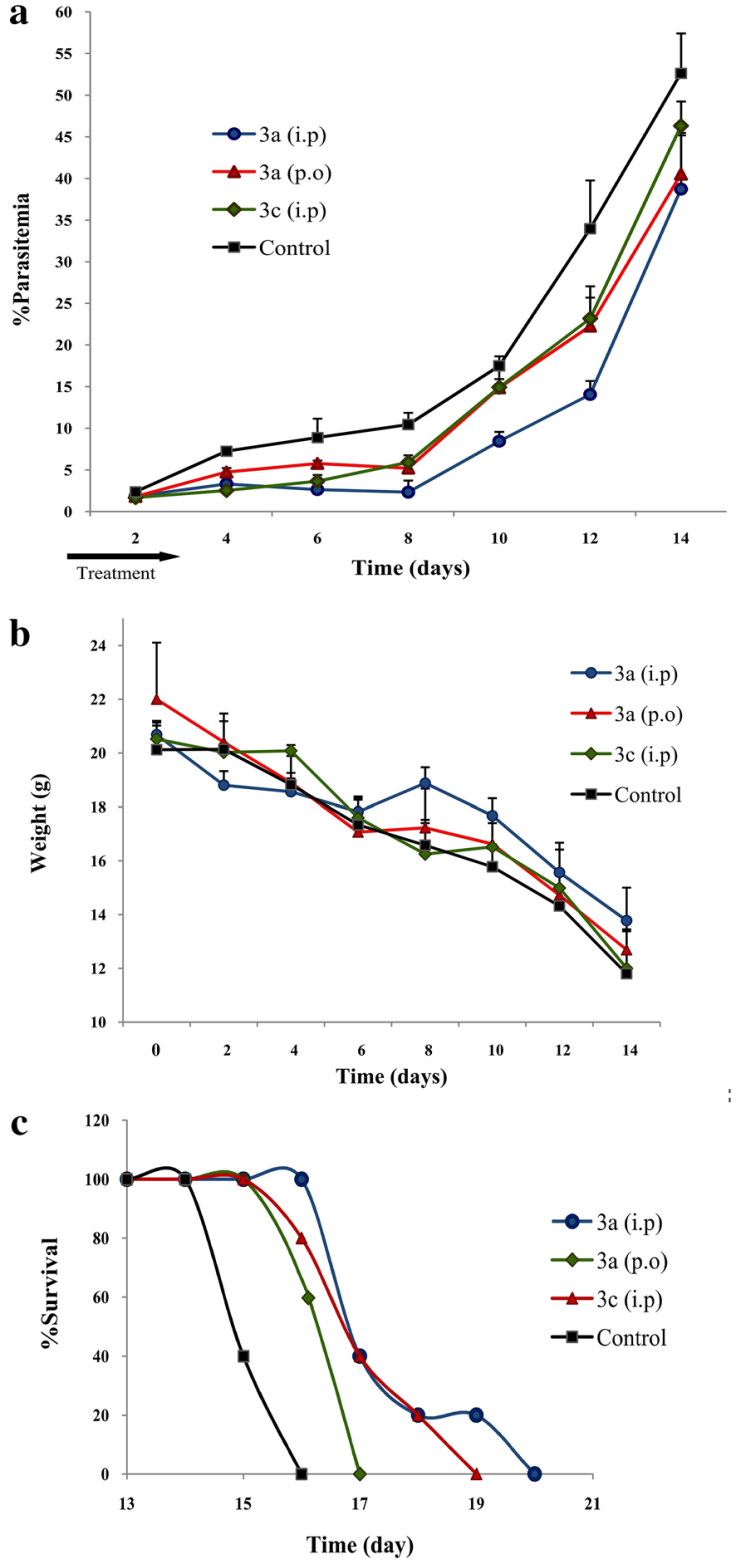
Pharmacodynamic profile in mice treated with compound **3** by i.p. and p.o. administration from 3rd day (D2) until 15th day (D14). **a**  %Parasitaemia of infected mice; **b** Body weight of *P. berghei* infected mice; **c** Mean survival time

Compound **3** showed high activity in the lowest dose on the fifth day (D4) in Peters’ test was chosen for further pharmacodynamic study by i.p. and p.o. administration. Therefore,  % parasitaemia was examined on the third day (D2) after inoculation and continued every 2 days of the interval until the parasitaemia level became more than 35%. As shown in Fig. 7a, compound **3** (31 mg/kg, i.p) showed the best  % parasitaemia in D4 (2.54%), while the highest concentration (125 mg/kg, i.p) showed the better result on the seventh (D6) and ninth (D8) days after treatment with 2.64 and 2.33% average parasitaemia, respectively. A gradual increase in parasitaemia was observed after D4 and D8 for **3c** and **3a** by i.p. administration, respectively (Fig. 7a). The median survival time was 17.8 (17 to 20) and 17.4 (16–19) days for **3a** and **3c**, respectively (Fig. 7c).

A single dose of p.o. administration resulted in a decline in parasitaemia until the 9^th^ day after the treatment. Then, the parasitaemia increased until reaching the experimental endpoints, with median survival times of 16.6 (16 to 17) days (Fig. 7c). Weight exchange was completely related to  % parasitaemia in the studied groups. The treated mice with **3c** had weight reduction from D6 after treatment, while the groups **3a** with i.p. and p.o. administration showed the gain of weight on D8 which was more prominent for group **3a** (125 mg/kg, i.p) (Fig. 7b).

### Statistical analysis

The variance between the groups was compared to within-group in Peters’ test which showed a significant difference between the three studied dose groups (*P *< 0.05). The statistical analysis of Rane’s test showed no significant difference between the three studied dose groups (*P *> 0.05). Also, the Tukey multiple comparisons for groups **1** and **2** showed no significant difference between the means (*P *> 0.05) which could confirm the results of variance analysis. Also, the comparison of the variance between the groups for survival day in Peters’ test showed a significant difference between groups **1c**, **2c**, **4c**, and the negative controls with CQ (*P *< 0.05), although CQ showed a significant difference with all groups except group **2a**, and **4b** with *P* value > 0.05 in Rane’s test.

## Discussion

The anti-plasmodial activity of the synthetic compounds (**1**–**4**) was evaluated against *P. berghei* with the suppressive and curative tests of Peters and of Rane [21, 23]. In the previous study, the compounds were tested against *P. falciparum* [Tahghighi et al. pers. commun]. The best result of PfLDH method was related to compound **1** with (nitrofuran-2-yl) methylene hydrazine side chain and 7-chloroquinoline ring that showed IC50 values of 0.3 and 0.6 µM against 3D7 and K1 strains (Additional file 1). Its thiophen analog (**2**) showed IC50 values of 0.5 and 0.6 µM against 3D7 and K1 strains, respectively. The compounds **1** and **2** were more active than the reference drug (CQ, IC50 = 0.7 µM) against K1 strain, but the compounds **3** and **4** with quinazoline ring displayed weaker results. The mode of action of CQ (Fig. 1) was associated with the interference with the detoxification of free haem in the lysosomal digestive vacuole of the parasite which was essential for its continuous growth and proliferation. Therefore, based on the structural similarity of the synthetic compounds with 4-aminoquinoline anti-malarials, their mode of action was evaluated by haemozoin formation assays in the previous study [Tahghighi et al. pers. commun]. The compounds (**1**–**2**), with the appropriate anti-plasmodial activity, showed higher inhibitory activity against haemozoin synthesis.

In the current study, the compounds was docked at the active site of the L-lactate dehydrogenase and haem in AutoDock Smina [18]. The best possible conformations with the lowest binding energies were identified (Table 1). The silico studies presented an effective and close binding energy toward the target proteins PfLDH and Haem ranging from − 8.0 to − 8.4 and − 5.1 to − 5.5 kcal/mol. Compound **2** showed five hydrogen binding interactions with active site amino acids Ile31, Thr97, Gly32, Lys51, Asp53, and Thr85 having energy − 8.0 kcal/mol exhibiting promising interaction on active site of PfLDH (Table 1, Fig. 2, Additional file 1). Based on the binding mode of ligands to haem, despite the insignificance of the differences, compound **1** and **3** have the highest binding affinity (Table 1, Fig. 3).

The compounds were studied at the concentrations of 125, 62, and 31 mg/kg for in vivo tests using mice inoculated with *P. berghei*. The oil and DMSO control groups showed higher parasitaemia than the studied compounds (Tables 2, 3 and Figs. 4, 5). Compound **2** in Peters’ test showed the highest  % suppression of parasitaemia (99.09%) on the 5 days (D4) after the treatment (Table 2, Fig. 4). Also, it was the most active compound in Rane’s test with 71.07% suppression of parasitaemia 5 days (D7) after the treatment (Table 3, Fig. 5). Notably, this compound had the highest lipophilicity among the four compounds studied and could easily cross the biological barriers (Table 3; ClogP = 4.46), while compound **1** showed the best result based on in vitro test (CLogP = 3.93). The comparison between the results of in vitro and in vivo tests verified the importance of in vivo test.

Compound **3**, with IC50 values of 2.8 and 3.7 µM against 3D7 and K1 strains, presented the best  % growth inhibition (78.74%) in the lowest dose (31 mg/kg) 5 days after the treatment (Table 2, Additional file 1). Whereas, the highest concentration (125 mg/kg) showed the best result 9 days after the treatment with 2.55% average parasitaemia (Table 2). For further evaluation, the pharmacodynamic study using the *P*. *berghei* murine malaria model was conducted. It confirmed that a dose of 31 mg/kg compound **3** had a faster initial decline in parasitaemia in three and five days after the treatment with 1.68 and 2.54% parasitaemia, respectively (Fig. 7a). But, dose 125 mg/kg following i.p. administration presented better inhibition of parasitaemia between D4 until D8 (3.32, 2.64. and 2.33, respectively) (Fig. 7a). Probably, long-period i.p. administration (125 mg/kg) can cause the complete decline of parasite.

While dose 125 mg/kg, p.o. administration had a partial change in percentage of parasitaemia between D6 until D8 (5.77 and 5.21, respectively) and showed lower parasitaemia compared with dose 31 mg/kg, i.p. on D8 with 5.91% parasitaemia (Fig. 7a). It is clear that p.o. administration had slow adsorption compared with i.p. administration. BALB/c mice were treated with 125 mg/kg, i.p. which had the lowest parasitaemia on 9th day also showed weight gain equivalent to 18.88 g (Fig. 7b). Also, mice with p.o. administration had partial weight gain equivalent 17.23 g on D8 that  % parasitaemia showed a decrease of 0.56%. Based on the pharmacodynamics profile, probably compound **3** had a slower absorption at the highest concentration, but over time it could slowly accumulate in the blood with the high concentration and kill parasites. On the 7th day of the study, the mice were dissected to compare the liver, spleen, and kidney with control groups in terms of toxicity (weight and discoloration). The drugs were dissolved in sesame oil. When dissected, those mice that received a dose of 125 of compound **3** had a completely oily abdomen. This means that the drug dissolved in the oil was still present in the peritoneal area and had not yet been fully absorbed into the blood. However, those mice that received a concentration of 31 mg/kg of this drug had no oily layer on their abdomens, indicating that the entire drug had been absorbed. Maybe at lower concentrations, the compound had a high initial uptake rate or was capable to eliminate the parasite in the blood before it entered the red blood cell, and after a few days due to its low concentration, the parasites would grow easily. The pharmacokinetic study must be performed in the next steps of the research to confirm this hypothesis. All synthetic compounds prolonged the mean survival time of treated mice in comparison with the negative control group (Fig. 7c).

Weight loss and discoloration of the internal organs on 7th day after the treatment can occur due to the toxicity of these compounds (Fig. 4) and maybe the lack of complete elimination of parasites during the treatment period. The anti-*Plasmodium* effect was also evaluated by Rane’s test. Compound **2** at the highest concentration (125 mg/kg) and compound **3** at the lowest concentration (31 mg/kg) showed the best  % inhibition of parasite growth with 71.07 and 79.42%, respectively on the eighth day after the treatment (Table 3). This test also emphasized that compound **3** had the best result at the beginning of treatment with the lowest concentration. Compound **2** (the best compound based on the Peters’ test) showed 71.07% of growth inhibition in the Rane’s test, where the treatment began 3 days after the inoculation of parasite and the parasite found an opportunity to grow (Tables 2 and 3). Chloroquine showed 96.10% suppression with a dose of 25 mg/kg and the mice survived longer than others.

Weight loss for treatment groups is induced by the lack of complete elimination of the parasites during the period of treatment which causes anaemia and anorexia. Based on the histopathological study, minimal congestion, vacuolization, and single-cell necrosis were observed in the liver of CQ-treated mice; however, they were not observed in the other groups which confirmed the fact that the target compounds were safe for the liver [24]. This requires more investigations in the future.

Although percentage parasitaemia decreased less than CQ, the need for a new, safe, well-tolerated, and low-cost alternative drug is seriously felt due to the spread of CQ resistance in many areas of the world. It seems that these compounds have the appropriate potential to replace quinoline anti-malarial drugs for simple synthesis, proper yield, and suitable anti-plasmodial activity.

## Conclusion

In this study, the results of in vivo tests verified that these compounds were effective on the blood stage of the malaria parasite. The results of docking studies corroborated that hydrogen bonding and several hydrophobic interactions between the ligands and the binding site of PfLDH and haem were responsible for the appropriate anti-plasmodial activity of the CQ analogs. Therefore, these compounds can be considered as new, effective CQ-analogs, especially in prevention. The compounds can be promising anti-malarial agents after further studies (e.g. formulation strategies, co-formulation with other anti-malarial drugs and the use of drug delivery systems in order to improve their pharmacokinetics).

## Supplementary information

**Additional file 1: Table S1.** In vitro anti-plasmodial activity of examined synthetic compounds against CQ-sensitive (3D7) and CQ-resistant (K1) *P. falciparum* strains. **Fig. S1.** The Ligplot analysis for L-lactate dehydrogenase and ligand interactions. Ligands are shown in magenta. Green dashed lines illustrate hydrogen bonds.

## Data Availability

All data generated or analysed during this study are included in this published article [and its additional file].

## Abbreviations

Ala: Alanine
ACT: Artemisinin-based combination therapy
ART: Artemisinin
Asp: Aspartic acid
BALB/c: Laboratory-bred strain of the house mouse
CQ: Chloroquine
Gly: Glycine
GMS: Greater Mekong Sub-region
IC50: Median Inhibitory Concentration
Ile: Isoleucine
JPEG: Joint Photographer’s Expert Group
i.p.: Intraperitoneal administration
1LDG: Crystal structure of PfLDH
Lys: Lysine
mg/kg: Milligram/kilogram
PfLDH: *Plasmodium falciparum* Lactate Dehydrogenase
PDB: Protein Data Bank
p.o.: Oral gavage administration
3P5Q: Crystal structures of haemoglobin
SPSS: Statistical Package for Social Sciences
Thr: Threonine
Tyr: Tyrosine
WHO: World Health Organization
