# Supplementary material for: In silico and in vivo anti-malarial investigation on 1-(heteroaryl)-2-((5-nitroheteroaryl)methylene) hydrazine derivatives
Source: Malar J. 2020 Jun 29;19:231. doi: 10.1186/s12936-020-03269-7 (PMC7322848; doi:10.1186/s12936-020-03269-7)
Supplement: Supplementary file 1 — Additional file 1: Table S1. In vitro anti-plasmodial activity of examined synthetic compounds against CQ-sensitive (3D7) and CQ-resistant (K1) P. falciparum strains. Fig. S1. The Ligplot analysis for L-lactate dehydrogenase and ligand interactions. Ligands are shown in magenta. Green dashed lines illustrate hydrogen bonds. [file 12936_2020_3269_MOESM1_ESM.docx]

**Table S1.** In vitro anti-plasmodial activity of examined synthetic compounds against CQ-sensitive (3D7) and CQ-resistant (K1) *P. falciparum* strains*

| **Compounds** | **pLDH assay IC_50_ (µM)Mean ± SD** | |
| --- | --- | --- |
|  | **3D7** | **K1** |
| **1** | 0.3 ± 0.1 | 0.6 ± 0.2 |
| **2** | 0.5 ± 0.2 | 0.6 ± 0.1 |
| **3** | 2.8 ± 0.6 | 3.7 ± 0.5 |
| **4** | 0.8 ± 0.2 | 0.9 ± 0.4 |
| **CQ** | 0.1 | 0.7 |

**IC_50_:** The half maximal inhibitory concentration

**CQ:** Chloroquine

**SD:** Standard deviation

*****Tahghighi et al. pers. commun


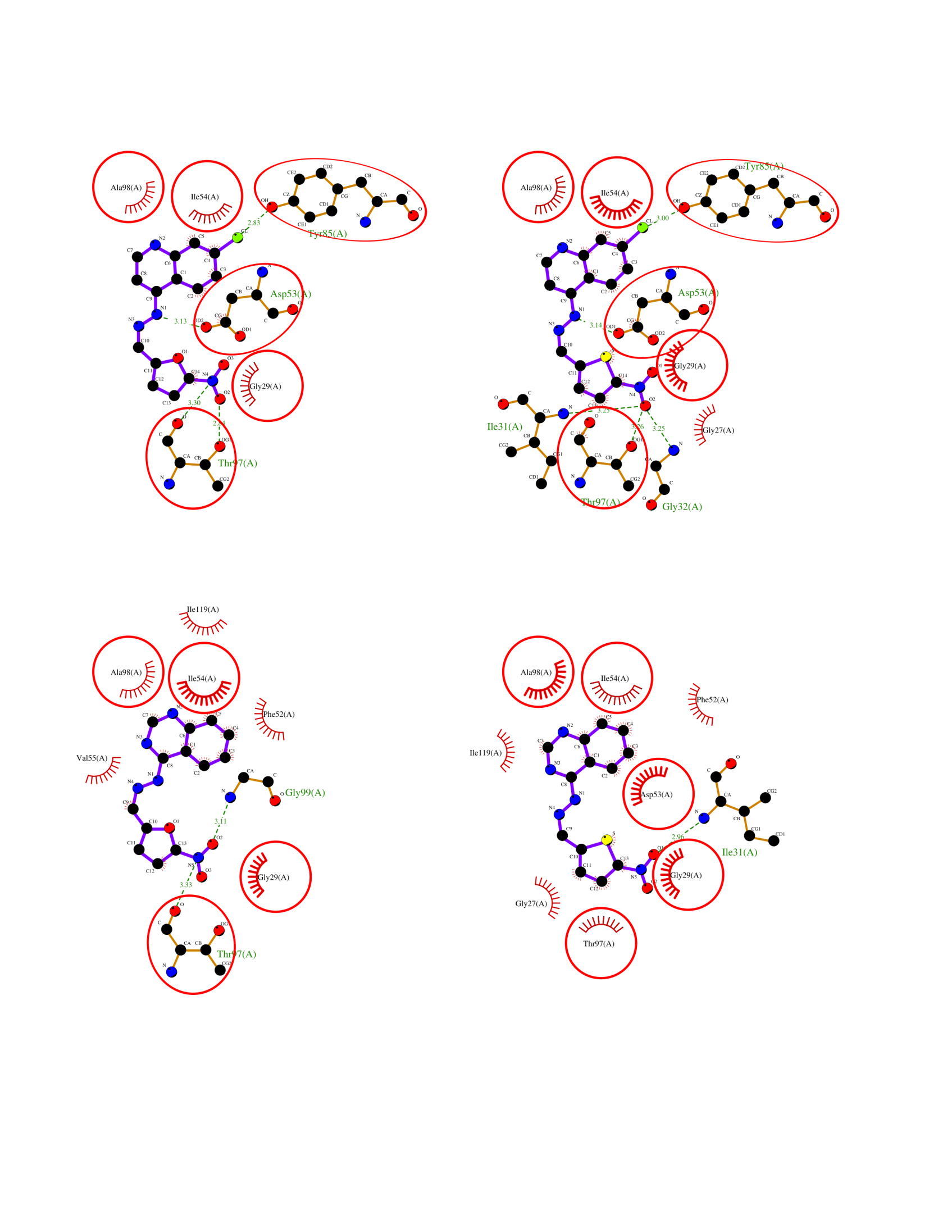


**Fig. S1 The Ligplot analysis for L-lactate dehydrogenase and ligand interactions.** Ligands are shown in magenta. Green dashed lines illustrate hydrogen bonds
